# Serum protein changes in a rat model of chronic pain show a correlation between animal and humans

**DOI:** 10.1038/srep41723

**Published:** 2017-02-01

**Authors:** Elisa Bellei, Antonietta Vilella, Emanuela Monari, Stefania Bergamini, Aldo Tomasi, Aurora Cuoghi, Simona Guerzoni, Letizia Manca, Michele Zoli, Luigi Alberto Pini

**Affiliations:** 1 Department of Diagnostic and Clinical Medicine and Public Health, Proteomic Lab, Center for Neuroscience and Neurotechnology, University of Modena and Reggio Emilia, via del Pozzo 71, 41124, Modena, Italy; 2 Department of Biomedical, Metabolic and Neural Sciences, Center for Neuroscience and Neurotechnology, University of Modena and Reggio Emilia, via Campi 287, 41125, Modena, Italy; 3 Science and Technology Park for Medicine, Mirandola, Modena, Italy

## Abstract

In previous works we showed the overexpression of some proteins in biological fluids from patients suffering chronic pain. In this proteomic study we analysed serum from a rat model of neuropathic pain obtained by the chronic constriction injury (CCI) of sciatic nerve, at two time intervals, 2 and 5 weeks after the insult, to find proteins involved in the expression or mediation of pain. Sham-operated and CCI rats were treated with saline or indomethacin. Two weeks after ligation, we identified three serum proteins overexpressed in CCI rats, two of which, alpha-1-macroglobulin and vitamin D-binding protein (VDBP), remained increased 5 weeks post-surgery; at this time interval, we found increased levels of further proteins, namely apolipoprotein A-I (APOA1), apolipoprotein E (APOE), prostaglandin-H2 D-isomerase (PTGDS) and transthyretin (TTR), that overlap the overexpressed proteins found in humans. Indomethacin treatment reversed the effects of ligation. The qPCR analysis showed that transcript levels of APOA1, APOE, PTGDS and VDBP were overexpressed in the lumbar spinal cord (origin of sciatic nerve), but not in the striatum (an unrelated brain region), of CCI rats treated with saline 5 weeks after surgery, demonstrating that the lumbar spinal cord is a possible source of these proteins.

Chronic pain (CP) arises from tissue injuries or inflammation, or from damages of the peripheral or central pain pathways (neuropathic pain). This latter is a disease state of the nervous system that can occur also in conditions in which there is no evident damage or inflammation (dysfunctional pain)^1^. CP is characterized by pain hypersensitivity, that is manifested by painful responses to normally non-painful stimuli (allodynia) or by increased or extreme sensitivity to pain stimuli (hyperalgesia), that can persist long time after the initial injury is resolved^2^. Nociceptor inputs can activate a prolonged but reversible increase in the excitability and synaptic efficacy of neurons in central nociceptive pathways, an event known as “central sensitization”, which manifests as pain hypersensitivity, tactile allodynia, pressure hyperalgesia and enhanced temporal summation^3^. Current treatment options for CP have often poor activity and undesirable side effects, hence there is an urgent need to better understand the molecular mechanisms of CP, in order to identify objective biomarkers to improve diagnosis, therapy and drug development for an effective treatment of pain^4^. In this context, proteomics can be employed to generate protein maps from different samples (including body fluids and tissues) and for correlating protein expression levels with the investigated disease^5^. Moreover, the discovery, design and evaluation of new drugs are strongly dependent on the elucidation of protein mechanisms involved in the respective disease. In particular, neuropathic pain reflects both peripheral and central sensitization mechanisms, which involve transcriptional and posttranscriptional modifications in sensory nerves^6^. Recently, metabolomics and proteomics studies from our and other groups have been applied in the attempt to identify new promising biomarkers and processes associated with CP^7^,^8^,^9^,^10^,^11^ and nerve regeneration^12^. A great advance in understanding the mechanisms that underlie CP has come from animal models of pain, which provide helpful and fundamental systems for preclinical pain studies^13^. A series of about 40 different animal models of neuropathic pain, each with distinct features and mechanisms, has been developed to mimic specific human pain conditions, by producing diseases or causing traumatic injuries to the spinal cord or peripheral nerves, that lead to painful states^14^. One of the most widely used model of chronic neuropathic pain is the unilateral sciatic nerve chronic constriction injury (CCI), developed in rats by *Bennet and Xie*^15^. It consists in four loose ligatures around the sciatic nerve, that occlude but do not arrest epineural blood flow. It is relatively simple to perform and produces robust and stable pain hypersensitivity for at least one month after injury. This model has a certain degree of variability due to rat gender, age, diet and strain, in addition to surgical variables, such as the tightness of the constrictions and the type of suture material used for nerve ligation^16^. Despite these limitations, numerous studies have proved the power of this model for the understanding of the neuropathic pain mechanisms.

Recently, innovative proteomic analysis concerning the expression of proteins in sciatic nerves of mice subjected to the CCI were performed by *Vacca et al*.^17^,^18^, demonstrating that the combination of proteomic methods with animal models of pain can help to identify pain-related proteins which may serve as diagnostic biomarkers or drug targets, improving the treatment of patients with CP^19^. In general, due to the incompleteness of genome sequencing for some animal species, the proteome analysis of most animals is still in the early stages, compared to the already well mapped human proteome. However, different methods are available to overcome this drawback, especially for the less characterized species, such as the homology search and the human annotation of BLAST-searched primary data^20^. However, this is not a concern for mouse and rat, since their genome has been completely sequenced, revealing a high homology with that of humans.

The objective of this study was to analyse, by a proteomic approach, serum samples from a rat model of neuropathic pain obtained by the CCI of sciatic nerve^15^, at two time intervals, 2 and 5 weeks after the insult. While these two intervals post-injury show similar signs of neuropathic pain^21^, they markedly differ as regards degenerative/regenerative processes of the sciatic nerve^22^. Indeed, sham-operated and CCI rats were treated with indomethacin between 2 and 5 weeks post-surgery, since it is a powerful medication^23^ and one of the most used non-steroidal anti-inflammatory drugs (NSAIDs) in human CP and chronic headaches^10^,^11^.

## Methods

### Animals and treatments

**Figure 1:**
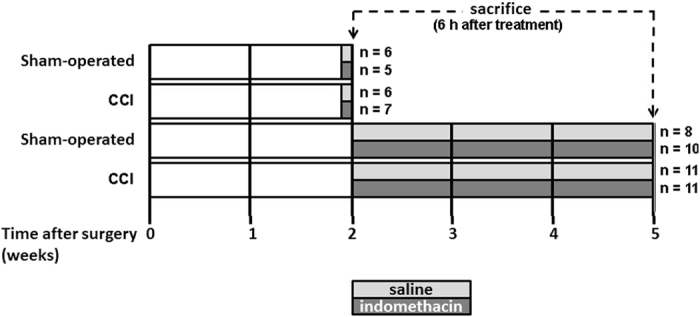
Treatment schedule. The number of rats/group is indicated. Light grey: saline treatment, dark grey: indomethacin treatment.

Adult male Wistar rats (Harlan Nossan, Italy) were matched for age (8 weeks) and weight (250–300 g). Eight groups of animals were considered (Fig. 1): four groups of rats were sacrificed 2 weeks after surgery: two sham-operated groups, treated once with saline (n = 6) or indomethacin (Liometacen, indomethacin meglumine salt, obtained from Alfa Wassermann, Pescara, Italy, 2 mg/kg *sc*) (n = 5), and two CCI groups, treated once with saline (n = 6) or indomethacin (n = 7). Four groups of rats were sacrificed 5 weeks after surgery, chronically treated for 21 days (from day 15 to day 35 post-surgery): two sham-operated groups chronically treated with saline (n = 8) or indomethacin (2 mg/kg *sc*/day) (n = 10), and two CCI groups chronically treated with saline (n = 11) or indomethacin (n = 11). All animals were sacrificed by decapitation at the same hour of the day to avoid the possibility that changes in protein expression levels can be attributed to circadian rhythms, 6 h after the last treatment. All efforts were made to minimize animal suffering and to reduce the number of rats employed.

Animals were housed in groups of two to four and inspected daily for infections or abnormal behaviour. They were allowed free access to both food and water for the entire duration of the experiments and housed under controlled temperature (22–25 °C) and humidity, with 12 h alternate light-dark cycles.

All animal care and experimental procedures were performed in strict accordance with the guidelines of the “Animal Care Committee” of the University of Modena and Reggio Emilia (Italy) that approved the study, and were in agreement with the directives of the “Italian Ministry of Health” (D.L. 116/92).

### Rat surgery

Rats were subjected to the CCI technique developed by Bennett and Xie^15^. The experimental procedure involved the unilateral ligation of the sciatic nerve at the high-thigh level of the right hind paw. Rats were deeply anesthetized using ketamine (110 mg/Kg, i.p.) and xilazine (3.6 mg/Kg, i.p.). The cutting area was first shaved and then, under sterile precautions, the sciatic nerve was exposed. Proximal to its trifurcation, 4 ligatures with 3-0 silk suture were tied loosely around the nerve at intervals of around 2 mm, so that the length of the treated nerve was approximately 8–10 mm, to preserve the epineurial circulation. The wound was closed with three external stitches and disinfected. Sham-operation consisted in anesthesia and surgery up to sciatic nerve exposure without ligation. Within few hours after the surgery, rats with sciatic nerve ligation developed spontaneous pain manifested by guarding behaviour of the operated paw and licking on the injury side. In the following days, they showed signs of paw lifting and limping, excessive grooming and biting, that have been suggested as nocifensive signs indicating the presence of spontaneous pain^24^. Moreover, rats with sciatic nerve ligation exibited signs of allodynia to mechanical stimulation assessed by Von Frey test 2 and 5 weeks after surgery, compared to sham-operated rats (Supplementary Fig. 1). Pain indicator signals persisted for the entire duration of the experiment. Autotomy was absent in all cases.

### Serum sample preparation for proteomic analysis

Blood was collected during animal decapitation into vacutainer serum separation tubes, and allowed to clot at room temperature for 1 h. Serum was separated by centrifugation at 2000 × g for 10 min at +4 °C. After the addition of a protease inhibitor cocktail (Sigma-Aldrich) to prevent protein enzymatic breakdown or modifications, samples were divided into aliquots and kept frozen at −80 °C until use. To reduce the complexity of serum samples before proteomic analysis, they were treated with the ProteoPrep^®^ Immunoaffinity albumin and IgG depletion kit (Sigma-Aldrich), that specifically removes the two most abundant serum proteins, albumin and IgG^25^. The protein content after depletion was assayed by the spectrophotometric Bradford’s method^26^, using bovine serum albumin (BSA) as standard.

### SDS-PAGE and two-dimensional gel electrophoresis

The proteomic analysis was performed on depleted serum samples, first by sodium dodecyl sulphate-polyacrylamide gel electrophoresis (SDS-PAGE) and then by two-dimensional gel electrophoresis (2-DE). SDS-PAGE was carried out according to Laemmli’s procedure^27^, under reducing conditions. Serum samples (20 μg of total proteins) were mixed with the 2× Laemmli sample buffer (Bio-Rad) supplemented with 0.5% dithiothreitol (DTT). Sample mixtures were boiled at 95 °C for 5 min, and then loaded onto 12% polyacrylamide gels. The electrophoresis was achieved using 1× Tris-glycine-SDS (TGS) running buffer pH 8.3 (Bio-Rad), initially at 100 V for 30 min, followed by an increase up to 200 V. Proteins were stained with colloidal Coomassie Blue G-250 by overnight incubation under gentle shaking, and finally destained with 10% methanol/5% acetic acid. Gels were done in triplicate and different percentage of acrylamide were tested to achieve the best resolution of protein bands. Gel images were acquired using a GS-800 calibrated densitometer (Bio-Rad) and analyzed by the “Quantity One 1-D Analysis Software” (version 4.6.7, Bio-Rad), as previously reported^28^. In brief, this software detects differentially expressed proteins among different groups on the basis of band staining intensity, converting signals from samples into digital data, which are displayed as a gray-scale. The total band intensity is expressed as optical density (OD).

Serum samples were subjected also to 2-DE analysis. Proteins (80 μg) were first diluted with rehydration buffer (6 M urea, 2 M thiourea, 4% CHAPS, 25 mM DTT, 0.2% ampholytes) and then loaded onto 7 cm immobilized pH gradient (IPG) strips, pH 3–10 (Ready Strip™, Bio-Rad). Proteins were separated by isoelectro focusing (IEF) at 20 °C, by an initial passive step of rehydration for 16 h, followed by a second IEF step at 500 V for 30 min, next ramping up to 5000 V for 5 h and finally focusing to reach 60000 V-h. Afterwards, the equilibration step and second-dimension separation were performed as previously reported in detail^28^. Finally, protein spots were visualized according to the Coomassie blue staining protocol described above. Gels were done at least in triplicate, testing different pH range of the strips and percentage of acrylamide in the second-dimension, to obtain the best spot resolution. Gel images were acquired by a calibrated densitometer and analyzed by the “PDQuest 2-D analysis software” program (version 7.3.1, Bio-Rad). This software compares bi-dimensional gel images to reveal increased or decreased protein spots based on their staining intensity, as previously described^10^. The abundance changes, both for the protein bands and for the spots, are shown in tables as fold-change of protein signal, calculated as ratio between the spot intensitiy values in CCI-saline or CCI-indomethacin *vs* sham-operated saline.

### Protein identification by mass spectrometry

The differential bands and spots were excised from the gels and subjected to an “in-gel” trypsin digestion protocol as previously reported^28^, prior to mass spectrometry (MS) analysis. The peptide mixtures were analyzed by a Nano LC-Chip-MS System, formed by the 6520 Accurate-Mass Quadrupole-Time of Flight Liquid Chromatography/Mass Spectrometry (Q-ToF LC/MS) coupled with a 1200 Nano HPLC-Chip microfluidic device (Agilent Technologies, CA, USA), as previously described^25^. Protein-identification peak lists were generated using MASCOT search engine (http://mascot.cigs.unimore.it/mascot), selecting the UniProtKB database.

### Quantitative real-time polymerase chain reaction

After animal decapitation, striatum and lumbar spinal cord were dissected out, lysed by mechanical disruption in Trizol reagent (Qiagen), homogenized following the procedure provided by the manufacturer (RNeasy Plus Mini Kit, Qiagen) and processed for quantitative PCR (qPCR) as described^29^. Isolated mRNA was reverse transcribed to cDNA using random hexamers and M-MLV Reverse Transcriptase (Promega Corporation) following the instructions provided by the manufacturer. Samples were heated at 70 °C for 5 min to eliminate any secondary structures, then incubated at 23 °C for 10 min, 1 h at 37 °C, and 5 min at 95 °C before being chilled at 4 °C using a thermocycler T Gradient (Whatman, Biometra). The amount of cDNA was quantified with iTaq Universal SYBR Green Supermix (Bio-Rad) using a Bio-Rad RT-PCR iCycler. Each PCR reaction was performed in triplicate using 150 nM of each primer, 10 μL of iTaq Universal SYBR Green Supermix (Bio-Rad), cDNA and nuclease-free water with the following cycling parameters: 10 min at 95 °C and 40 cycles of 1 min at 95 °C, 1 min at 60 °C and 1 min at 72 °C, followed by a melting curve analysis. RT-PCR primers were designed in two different exons; primer length was comprised between 18 and 30 bp, GC content was between 40 and 60% and nonspecific primer annealing and mismatches were minimized. The presence of nonspecific products of amplification and primer-dimers was evaluated by melting curve analysis during RT-PCR primer validation. The following primers were used to amplify the transcripts of interest:

GADPH Fw: 5′-CATCAAGAAGGTGGTGAAGC-3′

GADPH Rv: 5′-ACCACCCTGTTGCTGTAG-3′

PTGDS Fw: 5′-CAAGACAAGTTCCTGGGGCG-3′

PTGDS Rv: 5′-GTGCCAGACAGTGGTAGCTC-3′

TTR Fw: 5′-TCGATGTGGCCGTGAAAGTG-3′

TTR Rv: 5′-CGGAAGGGGTGTACAGGGTA-3′

APOE Fw: 5′-TTGGTCCCATTGCTGACAGG-3′

APOE Rv: 5′-GCGCAGGTAATCCCAGAAGC-3′

APOA1 Fw: 5′-TCTTCCTGACAGGTTGCCAAG-3′

APOA1 Rv: 5′-TGGCGAAATCCTTCACCCTG-3′

A1M Fw: 5′-GGACAGACAGTGAAATTCCGAG-3′

A1M Rv: 5′-GCAGTCCTCCTGGTAGATCG-3′

VDBP Fw: 5′-GGAAAGGAAAAATCAAGGATGAGCC-3′

VDBP Rv: 5′-ATGTGTGTTCAGGCAGCTCTC-3′

APOA4 Fw: 5′-CCAAGGAGGCTGTGGAACAA-3′

APOA4Rv: 5′-CACTCAGTTGAACGGCGAAG-3′

All the primers were shown to efficiently recognize their target mRNA using as positive controls spinal cord tissue (APOA1, APOE, PTGDS, VDBP), hypothalamus (TTR)^30^ or liver (APOA4, A1M).

Fold differences of expression were calculated using the comparative method, also referred as ΔΔCt method^29^. The sample of sham-operated saline was used as the reference sample or calibrator.

### Data analysis

All data are presented as mean ± standard error of the mean. Statistical comparisons were performed using the 2-way analysis of variance (ANOVA), considering a p-value ≤ 0.05 as statistically significant.

## Results

### SDS-PAGE analysis of serum proteins

**Figure 2:**
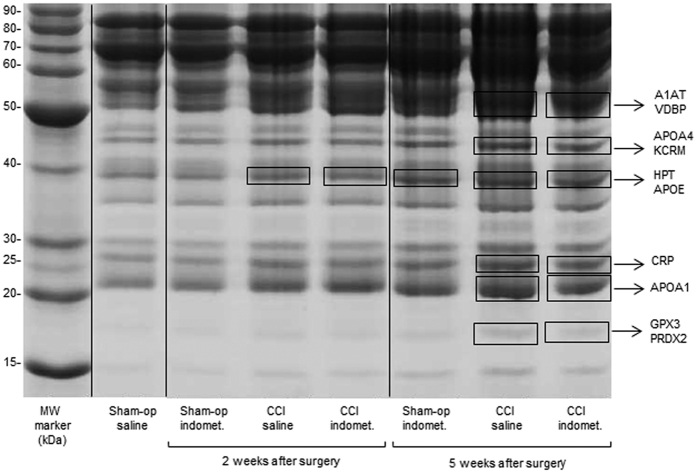
SDS-PAGE protein profile of depleted serum. The lanes report: MW marker, Molecular Weight protein ladder (BenchMark); Sham-op saline = sham-operated group treated with saline; sham-op indomet. = sham-operated group treated with indomethacin; CCI-saline = ligated group treated with saline; CCI-indomet. = ligated group treated with indomethacin, at 2 and 5 weeks post-surgery. The protein bands differentially expressed between the different groups analysed by Q-ToF LC/MS are enclosed in rectangles: A1AT: Alpha-1-antitrypsin, VDBP: Vitamin-D binding protein, APOA4: Apolipoprotein A-IV, KCRM: Creatine kinase M-type, HPT: Haptoglobin, APOE: Apolipoprotein E, CRP: C-reactive protein, APOA1: Apolipoprotein A-I, GPX3: Glutathione peroxidase 3, PRDX2: Peroxiredoxin-2.

The densitometric analysis by QuantityOne software revealed several bands differentially expressed (Fig. 2). These bands were cut from the gel and analysed by MS. Six protein bands appeared increased (2 to 5-fold) in the group of CCI rats treated with saline 5 weeks after the surgery compared to sham-operated rats treated with saline, whereas one of these bands was already increased in CCI rats 2 weeks after surgery. These changes were partially reversed by indomethacin treatment (Table 1).

The MS analysis of rat serum at 5 weeks post-surgery identified the following proteins, listed in Table 1: alpha-1-antitrypsin (A1AT), vitamin D-binding protein (VDBP), apolipoprotein A-IV (APOA4), creatine kinase M-type (KCRM), haptoglobin (HPT), apolipoprotein E (APOE), C-reactive protein (CRP), apolipoprotein A-I (APOA1), and 2 enzymes, glutathione peroxidase 3 (GPX3) and peroxiredoxin-2 (PRDX2). The protein band containing HPT and APOE was increased also in CCI rats treated with saline 2 weeks after surgery (Fig. 2, Table 1).

In Table 1, only the proteins showing the highest ion score and peptide match, as well as the greater sequence coverage obtained by MASCOT search engine (MS/MS ion search) through the UniProtKB database, are reported. The score is calculated as [−10 × log(*P*)], where *P* is the probability that the observed match between the experimental data and the database sequence is a random event; the peptide match refers to the total number of peptides that matched the identified protein; the sequence coverage indicates the percentage of amino acids sequenced for the detected protein. In addition, another parameter that was taken into account for protein selection was the MW; proteins with MW not conforming with those indicated by the MW marker standard were not considered.

### Two-dimensional gel electrophoresis of serum proteins

**Figure 3:**
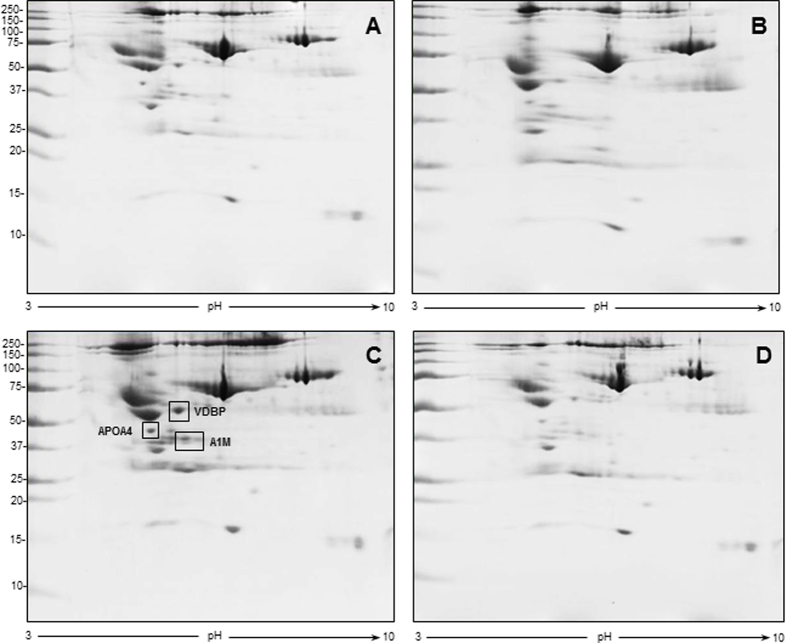
Representative 2D gel images for the 2 week time interval after surgery. First-dimension separation was obtained by IPG strip, pH range 3–10; second-dimension separation was achieved by 8–16% gradient gel. (**A**) sham-operated group treated with saline, (**B**) sham-operated group treated with indomethacin, (**C**) CCI-saline, (**D**) CCI-indomethacin. In panel C, rectangles indicate the increased proteins *vs* panel D, identified by MS and listed in Table 2 (VDBP: Vitamin D-binding protein, APOA4: Apolipoprotein A-IV, A1M: Alpha-1-macroglobulin).

**Figure 4:**
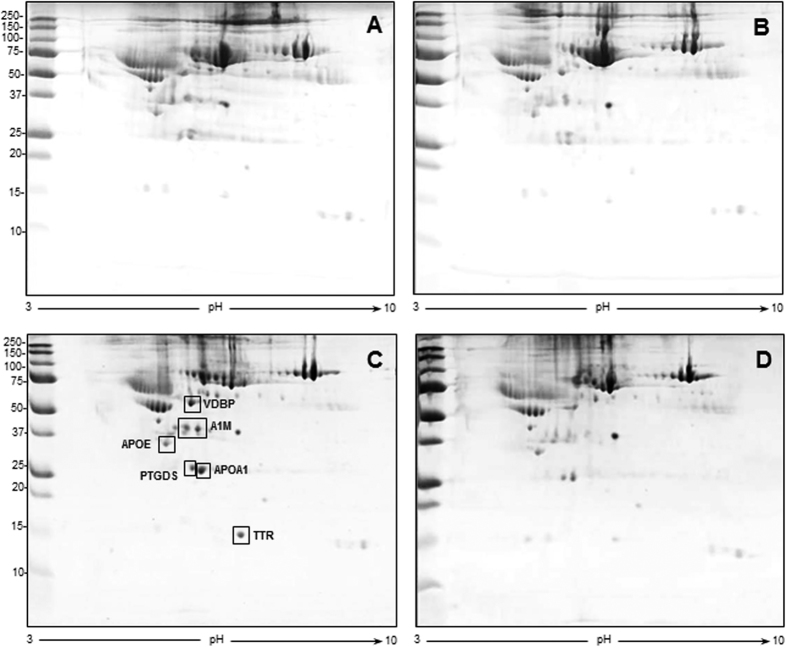
Representative 2D gel images for the 5 week time interval after surgery. First-dimension separation by IPG strip, pH range 3–10, second-dimension separation by 8–16% gradient gel. (**A**) sham-operated group treated with saline, (**B**) sham-operated group treated with indomethacin, (**C**) CCI-saline, (**D**) CCI-indomethacin. In panel C, rectangles indicate the increased proteins *vs* panel D, identified by MS and listed in Table 3 (VDBP: Vitamin D-binding protein, A1M: Alpha-1-macroglobulin, APOA1: Apolipoprotein A-I, PTGDS: Prostaglandin-H2 D-isomerase, TTR: Transthyretin, APOE: Apolipoprotein E).

The 2-DE coupled to MS analysis confirmed some of the data obtained by SDS-PAGE and permitted to identify and estimate by means of the PDQuest image analysis software (Bio-Rad) further differentially-expressed proteins at both 2 and 5 week time intervals (Figs 3 and 4, Table 2 and 3, respectively). At 2 weeks post-surgery, the level of 3 proteins was increased 9–10.5-fold in the group of CCI-saline compared to sham-operated rats treated with saline: VDBP, APOA4, and alpha-1-macroglobulin (A1M). Acute indomethacin treatment completely reversed this increase (Fig. 3 and Table 2).

At 5 weeks post-surgery, the levels of two of the proteins identified at 2 weeks post-surgery, VDBP and A1M, were still increased (about 8.5 and 6-fold increase, respectively) in CCI-saline with respect to sham-operated rats treated with saline. In addition, the levels of 4 additional proteins were increased in ligated rats treated with saline, corresponding to prostaglandin-H2 D-isomerase (PTGDS, 7.5-fold increase), transthyretin (TTR, 20-fold increase), APOA1 (22.5-fold increase) and APOE (3.3-fold increase), as illustrated in Fig. 4 and defined in Table 3. Chronic indomethacin treatment could substantially revert these increases with the exception of APOE whose levels remained elevated.

### Quantitative PCR analysis of central nervous tissues

**Figure 5:**
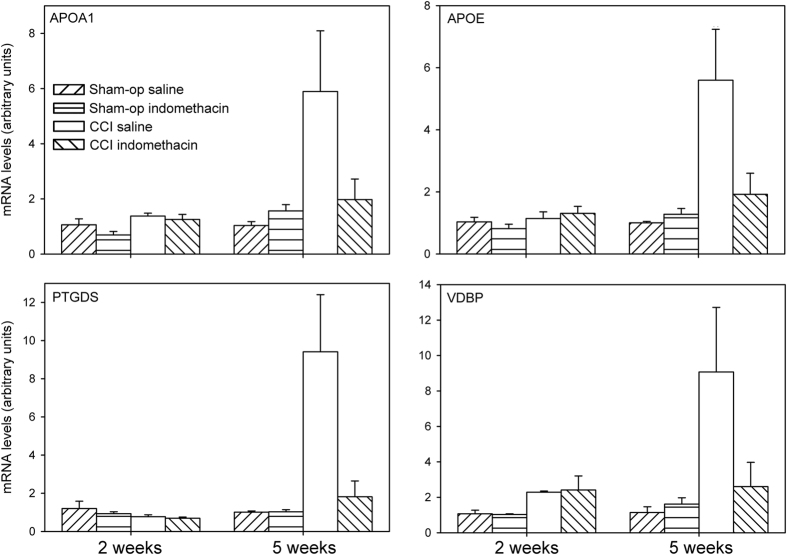
Levels of APOA1, APOE, PTGDS and VDBP mRNA in the lumbar spinal cord. The levels of APOA1, APOE, PTGDS and VDBP mRNA were measured in the lumbar spinal cord of rats, 2 or 5 weeks after a CCI of the sciatic nerve. The data were analysed by means of two-way ANOVA with sham-operation/ligation and saline/indomethacin as factors, showing a significant interaction for all 4 mRNAs in the 5 week groups.

Proteins whose levels are increased in the serum of ligated rats may derive from multiple sources in the body. A distinct possibility is that some of them derive from the lumbar spinal cord at the level of sciatic nerve root entry. To test this hypothesis, by using qPCR, we measured in the lumbar spinal cord the mRNA levels of 7 proteins whose levels were found to be altered in the serum of the same animals. Three of the transcripts, TTR, APOA4 and A1M, were not detected in the lumbar spinal cord. Instead, PTGDS, APOE, APOA1 and VDBP were detected in the extract from lumbar spinal cord and differentially expressed in the different treatment groups. As regards PTGDS, no significant difference was detected between the 2 week post-surgery treatment groups (two-way ANOVA, lesion F(1,11) = 2.051, p = 0.180; drug F(1,11) = 0.566, p = 0.467; lesion × drug F(1,11) = 0.161, p = 0.696), whereas, at 5 weeks post-surgery, a significant increase by about 9-fold was present in CCI rats chronically-treated with saline, that was markedly reduced by indomethacin treatment (two-way ANOVA, lesion F(1,18) = 6.486, p = 0.020; drug F(1,18) = 4.409, p = 0.050; lesion × drug F(1,18) = 4.455, p = 0.049) (Fig. 5).

A similar, though less intense effect (about 5-fold) was detected for APOE (two-way ANOVA, 2 week treatments: lesion F(1,11) = 0.996, p = 0.340; drug F(1,11) = 0.134, p = 0.721; lesion × drug F(1,11) = 1.136, p = 0.309; 5 week treatment: lesion F(1,18) = 9.952, p = 0.006; drug F(1,18) = 4.204, p = 0.057; lesion × drug F(1,18) = 5.690, p = 0.030) (Fig. 5).

A different pattern of effect was instead detected for APOA1 and VDBP mRNAs. At 2 weeks post-surgery, a significant, though modest, effect of ligation was detected that was not reverted by indomethacin treatment (two-way ANOVA, APOA1: lesion F(1,11) = 5.093, p = 0.045; drug F(1,11) = 1.587, p = 0.234; lesion × drug F(1,11) = 0.402, p = 0.539; VDBP: lesion F(1,11) = 5.016, p = 0.047; drug F(1,11) = 0.001, p = 0.970; lesion × drug F(1,11) = 0.009, p = 0.926). At 5 weeks post-surgery, significant increases by about 6- and 9-fold for APOA1 and VDBP, respectively, were present in CCI rats, that were reduced by indomethacin treatment (two-way ANOVA, APOA1: lesion F(1,18) = 6.384, p = 0.022; drug F(1,18) = 2.648, p = 0.123; lesion × drug F(1,18) = 4.569, p = 0.048; VDBP: lesion F(1,18) = 6.288, p = 0.024; drug F(1,18) = 2.836, p = 0.113; lesion × drug F(1,18) = 3.813, p = 0.070) (Fig. 5).

As a control, we measured the levels of the same 7 mRNAs in the striatum. In this region, 5 out of the 7 transcripts we have considered (TTR, APOA4, A1M, APOA1 and VDBP) were not detected. APOE and PTDGS were detected at levels, respectively, comparable to and lower than those of the lumbar spinal cord. As regards both APOE and PTGDS, no significant difference was detected between any treatment group 5 weeks after surgery (APOE: lesion F(1,23) = 0.786, p = 0.385; drug F(1,23) = 1.005, p = 0.326; lesion × drug F(1,23) = 0.009, p = 0.925; PTGDS: lesion F(1,22) = 0.554, p = 0.464; drug F(1,22) = 0.549, p = 0.467; lesion × drug F(1,22) = 0.193, p = 0.665) (Supplementary Fig. 2).

## Discussion

The main result of this study is the identification by means of a proteomic approach of several serum proteins that are markedly overexpressed in the CCI model of sciatic nerve ligation in the rat. Protein overexpression was partially different at 2 and 5 weeks post-surgery and was sensitive to indomethacin treatment. More specifically, A1M, APOA4 and VDBP levels were markedly increased 2 weeks after nerve ligation; while APOA4 levels declined to sham-operated control levels, A1M and VDBP levels remained increased at 5 weeks post-surgery. Moreover, at this latter time point, increased levels of a further set of proteins was detected: APOA1, APOE, PTGDS and TTR. In addition, we started a search for the possible source(s) in the body of serum proteins shown here to be overexpressed. We focused our investigation on the lumbar spinal cord, the site of origin and termination of the motor and sensory components, respectively, of the sciatic nerve, and the striatum, an unrelated brain region, where we measured by qPCR transcript levels of 7 proteins previously shown to be overexpressed at the proteomic analysis. Four of these transcripts, APOA1, APOE, PTGDS and VDBP, were expressed in the spinal cord and were markedly and selectively increased in CCI-saline 5 weeks after surgery. No significant change in any of the transcripts was observed in the striatum. The lumbar spinal cord is therefore a possible source of several proteins found increased in the serum of rats 5 weeks after sciatic nerve ligation.

Previous studies have shown that signs of hyperalgesia and alterations related to nerve degeneration/regeneration processes differ at different time intervals from sciatic nerve ligation. In the CCI model, signs of hyperalgesia such as duration of lesioned paw lifting in several pain tests (neutral plate, hot plate, pin prick, acetone spray or cold plate) as well as resistance to pressure at the Von Frey test, remained constant at 2 and 5 weeks post-ligation^21^, and were similar, or slightly lower, with respect to the first days post-surgery, indicating that hyperalgesia level is substantially maintained between 2 and 5 weeks post-ligation. On the other hand, the study of degenerative/regenerative processes of the ligated sciatic nerve showed a complex pattern of post-surgery changes^22^. Massive wallerian degeneration of mylelinated fibers, together with loss of unmyelinated fibers and synpathetic fibers, peaked during the second week post-ligation. While attenuated signs of nerve degeneration persisted in the following weeks up to 15 weeks post-ligation, starting from the third week signs of progressive nerve regeneration were detected, so that at 5 weeks post-ligation several nerve morphological features had partially recovered. Accordingly, sensory and motor nerve conduction velocities started to recover 2 weeks after the injury^31^. It is noteworthy that most instances of overexpression of serum protein and corresponding spinal mRNA levels were not observed at 2 weeks post-ligation, a period when the peak of degenerative processes was detected, but at 5 weeks post-ligation when intense signs of regeneration were (also) detected.

The proteins that we found overexpressed 2 and/or 5 weeks after sciatic nerve ligation have been previously implicated in pain, inflammatory or regenerative processes.

A1M belongs to the alpha macroglobulins family, a group of large glycoproteins that inhibit all types of proteinases by a trapping mechanism^32^. This protein was found overexpressed at both 2 and 5 weeks post-injury and may have different functions. In fact, it was found to be up-regulated in models of acute inflammation^33^,^34^,^35^ and has been involved in nerve regeneration, as it was shown to stimulate neurite outgrowth of embryonic cerebral cortical neurons *in vitro* and rat sciatic nerve regeneration *in vivo*, as well as nerve growth factor-promoted neurite outgrowth in pheochromocytoma PC12 cells^36^.

In CCI-rats we detected the overexpression of several apolipoproteins, i.e., APOA4, APOA1 and APOE. During nerve regeneration, large amounts of lipids are required for axonal regeneration and remyelination. It is thought that lipoproteins originating from axon and myelin breakdown in injured peripheral nerves supply cholesterol to regenerating axons^37^. Accordingly, increased levels of four apolipoproteins, including APOD, APOA4, APOA1 and APOE, were found in extracts of injured sciatic nerve, thus proving their accumulation in the regenerating peripheral nerves^12^,^38^,^39^. In our study, by 2-DE analysis we found increased serum levels of APOA4 selectively at 2 weeks after surgery, while APOA1 and APOE were found overexpressed selectively at 5 weeks after surgery. This is in broad accordance with *Jiménez et al*.^12^ who showed that APOA4 is transiently increased after injury, whereas APOA1 and APOE have a progressive increase up to 35 days after injury. As mentioned above, after nerve injury the request of lipids increases, and apolipoproteins from various sources play a pivotal role in the requested cholesterol transfer^39^. Probably, APOA4 is produced in the early stage of damage, during nerve degeneration in response to denervation. Afterwards, as remyelination and regeneration processes begin (in the second and third week after injury), and concurrently Schwann cells deplete their cholesterol stores^39^, the lipid transport system becomes more active. Consequently, further proteins increase, such as APOA1 and APOE. This latter is a 34 kDa glycoprotein known to influence degenerative, as well as regenerative events in the peripheral nervous system^40^. After peripheral nerve injury, axons degenerate and Schwann cells reabsorbe myelin; at the same time, APOE synthesis by resident and monocyte-derived macrophages and by endothelial cells increase greatly, leading to APOE accumulation within the nerve^39^,^41^,^42^. All these reports support the concept that APOE is involved in re-utilization of cholesterol from degenerating nerves for axonal repair and reconstruction of axonal and myelin membranes (regeneration process)^37^,^43^. These sets of evidence point to a potential role of APOE in functional recovery and/or reduction of neuropathic pain after peripheral nerve damage^44^.

TTR (also called prealbumin) is an ubiquitous protein, present in both the cerebrospinal fluid and serum, with a specific role in the transport of thyroid hormones and, indirectly, of retinol^45^; it is highly expressed in choroid plexus epithelial cells and has been detected in retina pigment epithelium and in the liver (UniProtKB database). TTR is not thought to be expressed by neurons (present data) (Allen Mouse Brain Atlas (ABA), http://mouse.brain-map.org)^46^, but is expressed by Schwann cells^47^. Increased levels of TTR in CCI-saline rats 5 weeks post-surgery may be related to regeneration processes. An involvement of TTR has been reported in sensorimotor, post-traumatic nerve fiber regeneration, axonal growth enhancement during peripheral nervous system regeneration, and neuroprotection in Alzheimer’s disease^48^,^49^,^50^.

Another protein overexpressed 5 weeks after surgery in CCI-saline rats is PTGDS; its overexpression may be particularly relevant as a pathogenic mechanism for production and/or maintenance of hyperalgesia. PTGDS, also known as β-trace protein, is a glutathione-independent prostaglandin D synthase that catalyzes the conversion of prostaglandin H2 (PGH2) to prostaglandin D2 (PGD2) and is involved in various physiological functions, such as vasodilation, thermoregulation, smooth muscle contraction and relaxation, sleep induction and sedation, regulation of immune response, mediation of cellular homeostasis and modulation of nociception^51^,^52^. PGD2 represents the most abundant prostanoid produced in CNS of mammals^53^. Some studies conducted on animal models have proved that prostaglandins play fundamental roles in central sensitization at spinal level, resulting in induction of hyperalgesia and cutaneous allodynia^54^,^55^. In particular, it has been reported that PTGDS-deficient mice lack tactile pain and allodynia^56^, and have reduced pain hypersensitivity^57^.

VDBP was overexpressed both at 2 and 5 weeks after nerve injury in CCI-saline *vs* sham-operated saline. VDBP is a major plasma constituent in a broad range of animal species. It is a monomeric glycoprotein synthesized and secreted by the liver, which transports vitamin D sterols and metabolites^58^. It has other functions, including the ability to bind monomers of actin with high affinity, sequestering them from polymerization, and to associate with membrane-bound immunoglobulins on the surface of B-lymphocytes^59^. Of relevance in the present context is the possible involvement of VDBD and vitamin D in nerve regeneration^60^,^61^ and pain mechanisms. In fact, vitamin D is thought to influence pain manifestation, playing a role in the aetiology and maintenance of CP states and associated comorbidities^62^. Interestingly, it has been demonstrated that vitamin D inadequacy is associated with medication refractory muscoloskeletal pain and neuromuscolar dysfunction among patients with CP^63^, and treatment with vitamin D can alleviate pain in a majority of the patients with vitamin D deficiency^64^. In addition, another study reported a possible relationship between dysfunction of VDBP and migraine attacks^65^.

To sum up, while the changes in A1M, TTR, APOA4, APOA1 and APOE may participate to nerve cell regeneration through local supplementation of lipids and other unknown mechanisms, alterations in VDBP and PTGDS may play a pathogenic role in the mechanisms of central pain hypersensitivity. In particular, spinal production of PGD2 is thought to cause hyperalgesia and allodynia^66^.

Previous proteomic studies in biological liquids, such as urine^10^,^11^, cerebrospinal fluid^9^,^67^, muscle interstitial fluid^68^ of subjects suffering CP reported increased levels of most of the proteins that we found overexpressed in the serum of rats 5 weeks after ligation, including PTGDS^9^,^11^,^67^, TTR^9^,^10^,^68^, APOE^9^, VDBP^67^ and APOA1^9^,^68^. This evidence points to CCI at late post-surgery time intervals as a highly relevant animal model of human CP states. Indeed, it suggests that proteomic markers found in human studies do not reflect hyperalgesic states *per se* but rather are associated to CP states where chronic degenerative and regenerative processes coexist in affected nerves. The isomorphism between animal model and human pathological state opens up the possibility to study in depth and mechanistically the origin and function of these proteins and attribute them a role as markers of specific features of CP and related pathologies.

Notably, an acute or chronic (3 weeks) treatment with indomethacin was able to reverse most of the observed changes in protein expression at 2 or 5 weeks post-surgery. This evidence suggests that the processes leading to the observed CCI-induced increases in serum proteins are controlled by molecular targets modulated by indomethacin, a powerful NSAID with non-selective inhibitory activity on cyclooxygenase 1 and 2^23^. Besides common NSAID-like effects, indomethacin is thought to have specific, though not well known, pharmacological actions as suggested by the existence of a set of diseases involving CP that are exquisitely responsive to indomethacin^69^. Comparison of several NSAIDs in the present paradigm may help define possible selective actions and targets of NSAIDs in CP states.

In conclusion, using an animal model of chronic neuropathic pain, this study demonstrates an increased expression of specific serum proteins, several of which were shown to be overexpressed in biological fluids of patients suffering CP. This close correspondence between proteins overexpressed in biological fluids of patients with CP and rats with chronic neuropathic pain opens up the possibility of an in depth study of the origin and pathophysiological significance of CP biomarkers.

## Additional Information

**How to cite this article**: Bellei, E. *et al*. Serum protein changes in a rat model of chronic pain show a correlation between animal and humans. *Sci. Rep.* **7**, 41723; doi: 10.1038/srep41723 (2017).

**Publisher's note:** Springer Nature remains neutral with regard to jurisdictional claims in published maps and institutional affiliations.

## Supplementary Material

Supplementary Information

## Figures and Tables

**Table 1:** Differentially expressed proteins revealed by SDS-PAGE and identified by Q-ToF LC/MS.

Entry name(a)	Protein full name	Gene name	Acc. number(b)	MW (kDa)(c)	Score(d)	Peptide Match(e)	Seq. cov(f).	emPAI(g)	Fold change(h)	Fold change(i)
***2 weeks after surgery***										
HPT	Haptoglobin	Hp	P06866	39.1	603	170	28%	1.65	+2.0	+1.3
APOE	Apolipoprotein E	Apoe	P02650	35.8	219	44	27%	0.30		
***5 weeks after surgery***										
VDBP	Vitamin D-binding protein	Gc	P04276	55.1	1568	128	61%	4.71	+2.2	+1.5
A1AT	Alpha-1-antiproteinase	Serpina1	P17475	46.3	4400	318	57%	11.8		
APOA4	Apolipoprotein A-IV	Apoa4	P02651	44.4	2450	132	76%	7.01	+2.9	+1.9
KCRM	Creatine kinase M-type	Ckm	P00564	43.2	1054	42	24%	1.09		
HPT	Haptoglobin	Hp	P06866	39.1	1477	156	43%	1.65	+3.2	+2.9
APOE	Apolipoprotein E	Apoe	P02650	35.8	382	43	44%	1.22		
CRP	C-reactive protein	Crp	P48199	25.7	3117	367	36%	1.35	+2.5	+2.1
APOA1	Apolipoprotein A-I	Apoa1	P04369	30.1	776	74	65%	6.34	+2.1	+1.5
GPX3	Glutathione peroxidase 3	Gpx3	P23764	25.6	833	77	43%	2.84	+5.0	+2.1
PRDX2	Peroxiredoxin-2	Prdx2	P35704	21.9	138	12	30%	0.53		

^(a)^Protein entry name from UniProt knowledge database (all with extension_RAT).

^(b)^Protein accession number (UniProtKB database).

^(c)^Theoretical protein molecular weight.

^(d)^The highest scores obtained with MASCOT search engine.

^(e)^Total number of peptides matching the identified protein.

^(f)^Percentage of amino acids sequenced for the detected protein.

^(g)^Exponentially modified protein abundance index.

^(h)^Fold-change: protein total band intensity change in CCI-saline *vs* sham-operated saline.

^(i)^Fold-change: protein total band intensity change in CCI-indomethacin *vs* sham-operated saline.

**Table 2:** Serum proteins differentially expressed 2 weeks post-surgery by 2-DE analysis in the group of CCI-saline and identified by Q-ToF LC/MS.

Entry name(a)	Protein full name	Acc. number(b)	Gene name	MW (Da)	Score(c)	Peptide match/sig. match(d)	Seq/ sig. seq(e)	Seq. cov(f).	emPAI(g)	Fold change(h)	Fold change(i)
VDBP	Vitamin D-binding protein	P04276	Gc	55106	2546	239/161	33/28	61%	7.09	+9.2	0.94
APOA4	Apolipoprotein A-IV	P02651	Apoa4	44429	3168	256/157	40/29	83%	14.27	+10.6	+1.03
A1M	Alpha-1-macroglobulin	Q63041	A1m	168388	4186	229/162	18/15	13%	2.39	+10.4	0.79

^(a)^Protein entry name (all with extension_RAT) from UniProtKB database, conforming to Fig. 3.

^(b)^Primary accession number from UniProtKB database.

^(c)^The highest scores by MASCOT search engine.

^(d)^Total number of matched peptides and the significant matches.

^(e)^Total number of sequences and the number of significant sequences.

^(f)^Percentage of amino acids sequenced for the detected protein.

^(g)^Exponentially modified protein abundance index.

^(h)^Protein spot intensity change in CCI-saline *vs* sham-operated saline.

^(i)^Protein spot intensity change in CCI-indomethacin *vs* sham-operated saline.

**Table 3:** Serum proteins differentially expressed 5 weeks post-surgery by 2-DE analysis in the group of CCI-saline and identified by Q-ToF LC/MS.

Entry name(a)	Protein full name	Acc. number(b)	Gene name	MW (Da)	Score(c)	Peptide match/sig. match(d)	Seq/ sig. seq(e)	Seq. cov(f).	emPAI(g)	Fold change(h)	Fold change(i)
VDBP	Vitamin D-binding protein	P04276	Gc	55106	642	52/37	14/10	30%	1.01	+8.5	0.96
A1M	Alpha-1-macroglobulin	Q63041	A1m	168388	4989	299/218	17/15	13%	0.62	+6.1	0.84
APOA1	Apolipoprotein A-I	P04639	Apoa1	30100	1083	90/69	23/20	59%	14.29	+22.6	+1.92
PTGDS	Prostaglandin-D H2-isomerase	P22057	Ptgds	21301	709	42/36	6/5	31%	3.71	+7.6	+1.45
TTR	Transthyretin	P02767	Ttr	15824	750	45/32	11/8	59%	7.42	+20.4(j)	no detect(k).
APOE	Apolipoprotein E	P02650	Apoe	35788	4187	230/182	27/20	64%	14.63	+3.3	+2.8

^(a)^Protein entry name (all with extension_RAT) from UniProtKB database, conforming to Fig. 4.

^(b)^Primary accession number from UniProtKB database.

^(c)^The highest scores by MASCOT search engine.

^(d)^Total number of matched peptides and the significant matches.

^(e)^Total number of sequences and the number of significant sequences.

^(f)^Percentage of amino acids sequenced for the detected protein.

^(g)^Exponentially modified protein abundance index.

^(h)^Protein spot intensity change in CCI-saline *vs* sham-operated saline.

^(i)^Protein spot intensity change in CCI-indomethacin *vs* sham-operated saline.

^(j)^Fold change was calculated towards CCI-indomethacin since sham-operated rats had no detectable spot.

^(k)^Fold change was not calculated since sham-operated rats had no detectable spot.

## References

[b1] Woolf C. J. What is this thing called pain? J Clin Invest 120, 3742–3744 (2010). 21041955 10.1172/JCI45178 PMC2965006

[b2] Pallen C. J. Breaking the pain connection. Nat Med 14, 1313–1315 (2008). 19057553 10.1038/nm1208-1313

[b3] Woolf C. J. Central sensitization: implications for the diagnosis and treatment of pain. Pain 152, S2–S15 (2011). 20961685 10.1016/j.pain.2010.09.030 PMC3268359

[b4] Borsook D., Becerra L. & Hargreaves R. Biomarkers for chronic pain and analgesia. Part 1: the need, reality, challenges, and solutions. Discov Med 11(58), 197–207 (2011). 21447279

[b5] Hanash S. Disease proteomics. Nature 422, 226–232 (2003). 12634796 10.1038/nature01514

[b6] Woolf C. J. & Mannion R. J. Neuropathic pain: aetiology, symptoms, and management. Lancet 353, 1959–1964 (1999). 10371588 10.1016/S0140-6736(99)01307-0

[b7] Patti G. J. . Metabolomics implicates altered sphingolipids in chronic pain of neuropathic origin. Nat Chem Biol 8, 232–234 (2012). 22267119 10.1038/nchembio.767 PMC3567618

[b8] Hadrévi J. . Comparative metabolomics of muscle interstitium fluid in human trapezius myalgia: an *in vivo* microdialysis study. Eur J Appl Physiol 113, 2977–2989 (2013). 24078209 10.1007/s00421-013-2716-6 PMC3828502

[b9] Bäckryd E., Ghafouri B., Carlsson A. K., Olausson P. & Gerdle B. Multivariate proteomic analysis of the cerebrospinal fluid of patients with peripheral neuropathic pain and healthy controls – a hypothesis-generating pilot study. J Pain Res 8, 321–333 (2015). 26170714 10.2147/JPR.S82970 PMC4492642

[b10] Bellei E. . Discovery by a proteomic approach of possible early biomarkers of drug-induced nephrotoxicity in medication-overuse headache. J Headache Pain 14, 6 (2013). 23565828 10.1186/1129-2377-14-6 PMC3606963

[b11] Bellei E. . Validation of potential candidate biomarkers of drug-induced nephrotoxicity and allodynia in medication-overuse headache. J Headache Pain 16, 77 (2015). 10.1186/s10194-015-0559-8 PMC4536253 26272683

[b12] Jiménez C. R. . Proteomics of the injured rat sciatic nerve reveals protein expression dynamics during regeneration. Mol Cel Proteomics 4, 120–132 (2005). 10.1074/mcp.M400076-MCP200 15509515

[b13] Wang L. X. & Wang Z. J. Animal and cellular models of chronic pain. Adv Drug Deliver Rev 55, 949–965 (2003). 10.1016/s0169-409x(03)00098-x 12935939

[b14] Jaggi A. S., Jain V. & Singh N. Animal models of neuropathic pain. Fund Clin Pharmacol 25, 1–28 (2011). 10.1111/j.1472-8206.2009.00801.x 20030738

[b15] Bennett G. J. & Xie Y. K. A peripheral mononeuropathy in rat that produces disorders of pain sensation like those seen in man. Pain 33(1), 87–107 (1988). 2837713 10.1016/0304-3959(88)90209-6

[b16] Austin P. J., Wu A. & Moalem-Taylor G. Chronic constriction of the sciatic nerve and pain hypersensitivity testing in rats. J Vis Exp (61), e3393 (2012). 10.3791/3393 PMC3399467 22433911

[b17] Vacca V. . Higher pain perception and lack of recovery from neuropathic pain in females: a behavioural, immunohistochemical, and proteomic investigation on sex-related differences in mice. Pain 155, 388–402 (2014). 24231652 10.1016/j.pain.2013.10.027

[b18] Vacca V. . 17beta-estradiol counteracts neuropathic pain: a behavioural, immunohistochemical, and proteomic investigation on sex-related differences in mice. Sci Rep 6, 18980 (2016). 26742647 10.1038/srep18980 PMC4705539

[b19] Niederberger E. & Geisslinger G. Proteomics in neuropathic pain research. Anesthesiology 108, 314–323 (2008). 18212577 10.1097/01.anes.0000299838.13368.6e

[b20] Di Girolamo F. . Farm animal serum proteomics and impact on human health. Int J Mol Sci 15, 15396–15411 (2014). 25257521 10.3390/ijms150915396 PMC4200749

[b21] Dowdall T., Robinson I. & Meert T. F. Comparison of five different rat models of peripheral nerve injury. Pharmacol Biochem Behav 80, 93–108 (2005). 15652385 10.1016/j.pbb.2004.10.016

[b22] Guilbaud G. . Time course of degeneration and regeneration of myelinated nerve fibres following chronic loose ligatures of the sciatic nerve: can nerve lesions be linked to the abnormal pain-related behaviours? Pain 53(2), 147–158 (1993). 8393169 10.1016/0304-3959(93)90074-Y

[b23] Lucas S. The pharmacology of indomethacin. Headache 56, 436–546 (2016). 26865183 10.1111/head.12769

[b24] Bateson P. Assessment of pain in animals. Anim Behav 42, 827–839 (1991).

[b25] Bellei E. . High-abundance proteins depletion for serum proteomic analysis: concomitant removal of non-targeted proteins. Amino Acids 40, 145–156 (2011). 20495836 10.1007/s00726-010-0628-x

[b26] Bradford M. M. A rapid and sensitive method for the quantitation of microgram quantities of protein utilizing the principle of protein-dye binding. Anal Biochem 72, 248–254 (1976). 942051 10.1016/0003-2697(76)90527-3

[b27] Laemmli U. K. Cleavage of structural proteins during the assembly of the head of bacteriophage T4. Nature 227, 680–685 (1970). 5432063 10.1038/227680a0

[b28] Bellei E. . Proteomic analysis of urine in medication-overuse headache patients: possible relation with renal damages. J Headache Pain 13, 45–52 (2012). 21997203 10.1007/s10194-011-0390-9 PMC3253154

[b29] Ferretti S., Fornari A., Pedrazzi P., Pellegrini M. & Zoli M. Developmental overfeeding alters hypothalamic neuropeptide mRNA levels and response to a high-fat diet in adult mice. Peptides 32(7), 1371–1383 (2011). 21683751 10.1016/j.peptides.2011.06.001

[b30] Zheng F., Kim Y. J., Moran T. H., Li H. & Bi S. Central transthyretin acts to decrease food intake and body weight. Sci Rep 7(6), 24238 (2016). 10.1038/srep24238 PMC4823743 27053000

[b31] Bai Y. H., Takemitsu M., Atsuta Y. & Matsuno T. Peripheral mononeuropathy induced by loose ligation of the sciatic nerve in the rat: behavioral, electrophysiological and histopathologic studies. Exp Anim 48, 87–94 (1999). 10374069 10.1538/expanim.48.87

[b32] Xiao T., Decamp D. L. & Sprang S. R. Structure of a rat α_1_-macroglobulin receptor-binding domain dimer. Protein Sci 9, 1889–1897 (2000). 11106161 10.1110/ps.9.10.1889 PMC2144472

[b33] Ebrini I. . Proteins of rat serum V: adjuvant arthritis and its modulation by nonsteroidal anti-inflammatory drugs. Electrophoresis 21(11), 2170–2179 (2000). 10892728 10.1002/1522-2683(20000601)21:11<2170::AID-ELPS2170>3.0.CO;2-1

[b34] Ivanović Matić S. . Acute-phase protein expression in DMSO-intoxicated rats. Toxicol Lett 147(2), 153–159 (2004). 14757319 10.1016/j.toxlet.2003.11.011

[b35] Jain M. R. . Altered proteolytic events in experimental autoimmune encephalomyelitis discovered by iTRAQ shotgun proteomics analysis of spinal cord. Proteome Sci 16, 7:25 (2009). 10.1186/1477-5956-7-25 PMC2716311 19607715

[b36] Lee P. G. & Koo P. H. Rat α_1_-macroglobulin enhances nerve growth factor-promoted neurite outgrowth, TrkA phosphorylation, and gene expression of pheochromocytoma PC12 cells. J Neurochem 74, 81–91 (2000). 10617108 10.1046/j.1471-4159.2000.0740081.x

[b37] Posse de Chaves E. I., Vance D. E., Campenot R. B., Kiss R. S. & Vance J. E. Uptake of lipoproteins for axonal growth of sympathetic neurons. J Biol Chem 275, 19883–19890 (2000). 10867025 10.1074/jbc.275.26.19883

[b38] Boyles J. K., Notterpek L. M. & Anderson L. J. Accumulation of apolipoproteins in the regenerating and remyelinating mammalian peripheral nerve. J Biol Chem 265, 17805–17815 (1990). 2120218

[b39] Boyles J. K. . A role for apolipoprotein E, apolipoprotein A-I, and low density lipoprotein receptors in cholesterol transport during regeneration and remyelination of the rat sciatic nerve. J Clin Invest 83, 1015–1031 (1989). 2493483 10.1172/JCI113943 PMC303779

[b40] Comley L. H. . ApoE isoform-specific regulation of regeneration in the peripheral nervous system. Human Mol Gen 22, 2406–2421 (2011). 10.1093/hmg/ddr147 PMC3098734 21478199

[b41] Ignatius M. J. . Expression of apolipoprotein E during nerve degeneration and regeneration. P Natl Acad Sci USA 83, 1125–1129 (1986). 10.1073/pnas.83.4.1125 PMC323024 2419900

[b42] LeBlanc A. C. & Poduslo J. F. Regulation of apolipoprotein E gene expression after injury of the rat sciatic nerve. J Neurosci Res 25, 162–171 (1990). 2319626 10.1002/jnr.490250203

[b43] Li F. Q., Fowler K. A., Neil J. E., Colton C. A. & Vitek M. P. An apolipoprotein E-mimetic stimulates axonal regeneration and remyelination after peripheral nerve injury. J Pharmacol Exp Ther 334, 106–115 (2010). 20406857 10.1124/jpet.110.167882 PMC2912037

[b44] Melemedjian O. K., Yassine H. N., Shy A. & Price T. J. Proteomic and functional annotation analysis of injured peripheral nerves reveals ApoE as a protein upregulated by injury that is modulated by metformin treatment. Mol Pain 9, 14 (2013). 23531341 10.1186/1744-8069-9-14 PMC3623807

[b45] Dickson P. W., Aldred A. R., Marley P. D., Bannister D. & Schreiber G. Rat choroid plexus specializes in the synthesis and the secretion of transthyretin (Prealbumin). J Biol Chem 261, 3475–3478 (1986). 3949774

[b46] Sousa M. M. & Saraiva M. J. Transthyretin is not expressed by dorsal root ganglia cells. Exp Neurol 214(2), 362–365 (2008). 18835560 10.1016/j.expneurol.2008.08.019

[b47] Murakami T., Ohsawa Y., Zhenghua L., Yamamura K. & Sunada Y. The transthyretin gene is expressed in Schwann cells of peripheral nerves. Brain Res 1348, 222–225 (2010). 20547140 10.1016/j.brainres.2010.06.017

[b48] Mar F. M., Franquinho F., Fleming C. E. & Sousa M. M. Transthyretin in peripheral nerve regeneration. Future Neurol 4, 723–730 (2009).

[b49] Fleming C. E., Mar F. M., Franquinho F. & Sousa M. M. Transthyretin: an enhancer of nerve regeneration. Int Rev Neurobiol 87, 337–346 (2009). 19682646 10.1016/S0074-7742(09)87017-7

[b50] Fleming C. E., Saraiva M. J. & Sousa M. M. Transthyretin enhances nerve regeneration. J Neurochem 103, 831–839 (2007). 17897357 10.1111/j.1471-4159.2007.04828.x

[b51] Sorrentino C. . Rat prostaglandin D_2_ synthetase: its tissue distribution, changes during maturation, and regulation in the testis and epididymis. Biol Reprod 59, 843–853 (1998). 9746734 10.1095/biolreprod59.4.843

[b52] Urade Y. & Hayaishi O. Biochemical, structural, genetic, physiological, and pathophysiological features of lipocalin-type prostaglandin D synthase. Biochim Biophys Acta 1482, 259–271 (2000). 11058767 10.1016/s0167-4838(00)00161-8

[b53] Urade Y., Eguchi N. & Hayaishi O. *Lipocalin-type prostaglandin D synthase as an enzymic lipocalin*, In Lipocalins (ed. Akerstrom B., Borregaard N., Flower D. R. & Salier J. P.) 99–109 (Landes Bioscience, 2006).

[b54] Ito S., Okuda-Ashitaka E. & Minami T. Central and peripheral roles of prostaglandins in pain and their interactions with novel neuropeptides nociception and nocistatin. Neurosci Res 41, 299–332 (2001). 11755218 10.1016/s0168-0102(01)00289-9

[b55] Kawabata A. Prostaglandin E_2_ and pain – An update. Biol Pharm Bull 34, 1170–1173 (2011). 21804201 10.1248/bpb.34.1170

[b56] Eguchi N. . Lack of tactile pain (allodynia) in lipocalin-type prostaglandin D synthase-deficient mice. P Natl Acad Sci USA 96, 726–730 (1999). 10.1073/pnas.96.2.726 PMC15204 9892701

[b57] Kamei D. . Reduced pain hypersensitivity and inflammation in mice lacking microsomal prostaglandin E synthase-1. J Biol Chem 279, 33684–33695 (2004). 15140897 10.1074/jbc.M400199200

[b58] Ray K., Wang X., Zhao M. & Cooke N. E. The rat vitamin D binding protein (Gc-globulin) gene. J Biol Chem 266, 6221–6229 (1991). 2007578

[b59] Cooke N. E. Rat vitamin D binding protein. J Biol Chem 261, 3441–3450 (1986). 2419332

[b60] Chabas J. F. . Cholecalciferol (vitamin D_3_) improves myelination and recovery after nerve injury. PLoS One 8(5), e65034 (2013). 23741446 10.1371/journal.pone.0065034 PMC3669361

[b61] Chabas J. F. . Vitamin D2 potentiates axon regeneration. J Neurotrauma 25(10), 1247–1256 (2008). 18986226 10.1089/neu.2008.0593

[b62] Shipton E. A. & Shipton E. E. Vitamin D and pain: vitamin D and its role in the aetiology and maintenance of chronic pain states and associated comorbidities. Pain Res Treat 2015, ID904967 (2015). 10.1155/2015/904967 PMC4427945 26090221

[b63] Turner M. K. . Prevalence and clinical correlates of vitamin D inadequacy among patients with chronic pain. Pain Med 9, 979–984 (2008). 18346069 10.1111/j.1526-4637.2008.00415.x

[b64] Abbasi M., Hashemipour S., Hajmanuchehri F. & Kazemifar M. Is vitamin D deficiency associated with non specific musculoskeletal pain? Glob J Health Sci 5, 107–111 (2013). 23283042 10.5539/gjhs.v5n1p107 PMC4776981

[b65] Nagata E. . Possible association between dysfunction of vitamin D binding protein (Gc globulin) and migraine attacks. PLoS One 9, e105319 (2014). 25147936 10.1371/journal.pone.0105319 PMC4141767

[b66] Minami T. . Allodynia evoked by intathecal administration of prostaglandin E2 to conscious mice. Pain 57(2), 217–223 (1994). 7916452 10.1016/0304-3959(94)90226-7

[b67] Patton S. M. Proteomic analysis of the cerebrospinal fluid of patients with restless legs syndrome/Willis-Ekbom disease. Fluids Barriers CNS 10, 20 (2014). 10.1186/2045-8118-10-20 PMC3680184 23758918

[b68] Olausson P., Gerdle B., Ghafouri N., Larsson B. & Ghafouri B. Identification of proteins from interstitium of trapezius muscle in women with chronic myalgia using microdialysis in combination with proteomics. PLoS One 7(12), e52560 (2012). 23300707 10.1371/journal.pone.0052560 PMC3531451

[b69] VanderPluym J. Indomethacin-responsive headaches. Curr Neurol Neurosci Rep 15, 516 (2015). 25467407 10.1007/s11910-014-0516-y

