# Supplementary material for: Serum protein changes in a rat model of chronic pain show a correlation between animal and humans
Source: Sci Rep. 2017 Feb 1;7:41723. doi: 10.1038/srep41723 (PMC5286399; doi:10.1038/srep41723)
Supplement: Supplementary Information [file srep41723-s1.pdf]

Supplementary Information

Manuscript Title: **Serum protein changes in a rat model of chronic pain show a correlation between animal and humans.**

Author list: Elisa Bellei, Antonietta Vilella, Emanuela Monari, Stefania Bergamini, Aldo Tomasi[1], Aurora Cuoghi, Simona Guerzoni, Letizia Manca, Michele Zoli, Luigi Alberto Pini

Supplementary figure 1

Allodynia in sham-operated rats and CCI rats 2 or 5 weeks after surgey evaluated by means of the Von Frey test. All CCI rats showed signs of allodynia at both 2 and 5 weeks post-surgery.

Statistical analysis according to two-way ANOVA.

2 weeks: CCI/sham, $F(1,35) = 4.377$, $p=0.044$; saline/indomethacin, $F(1,35) = 1.342$, $p=0.255$; interaction, $F(1,35) = 1.464$, $p=0.234$.

5 weeks: CCI/sham, $F(1,30) = 10.096$, $p=0.003$; saline/indomethacin, $F(1,35) = 1.400$, $p=0.246$; interaction, $F(1,35) = 2.213$, $p=0.147$.

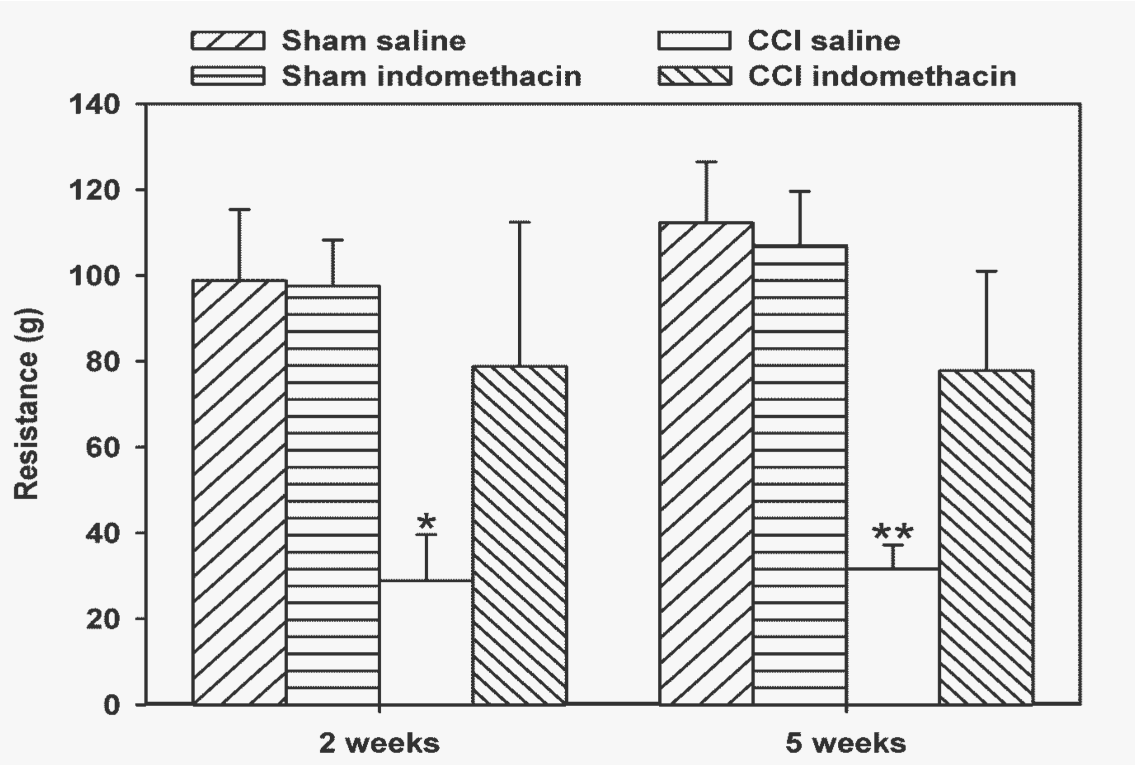

Supplementary figure 2

Levels of APOE and PTGDS mRNA in the striatum of rats 5 weeks after sham-operation or a CCI of the sciatic nerve.

The data were analysed by means of two-way ANOVA with sham-operation/ligation and saline/indomethacin as factors, showing no significant effect.

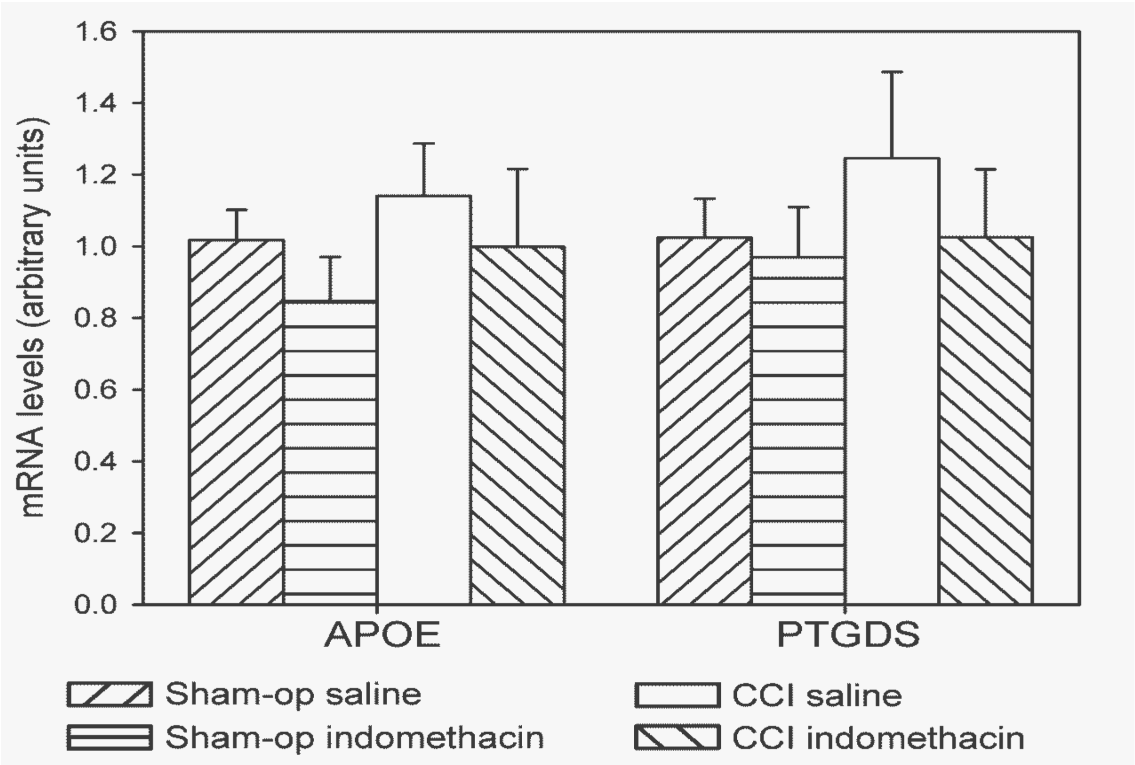

Supplementary method

Von Frey hair test. Mechanical sensitivity (allodynia) was assessed by Von Frey hair test, based on the responsiveness of the operated hind paw to the application of a series of von Frey filaments (Aesthesio®, USA), progressively increasing the stifness (ranging from 0.008 to 300 g). Animals were placed in a plexiglass cage with a metal grid floor, allowing them to move freely. Filaments were applied to the mid-plantar surface of both the operated and non-injured hindpaw. The smallest filament that elicited a foot withdrawal response was considered the threshold stimulus. Withdrawal thresholds at the contralateral (non-injured) side remained constant throughout the experiments.
